# Predictability of a modified Mini- Nutritional- Assessment version on six-month and one-year mortality in hospitalized geriatric patients: a comparative analysis

**DOI:** 10.1038/s41598-019-45452-0

**Published:** 2019-06-21

**Authors:** Lea Becker, Dorothee Volkert, Cornel Christian Sieber, Karl-Günter Gaßmann, Martin Ritt

**Affiliations:** 1 Department of Internal Medicine III (Medicine of Ageing), Geriatrics Centre Erlangen, Malteser Waldkrankenhaus St. Marien, Rathsberger Straße 57, D-91054 Erlangen, Germany; 2 Institute of Biomedicine of Ageing (IBA), Friedrich-Alexander University Erlangen-Nürnberg (FAU), Kobergerstraße 60, D-90408 Nürnberg, Germany; 3 Department of Internal Medicine and Geriatrics, Hospital of the Order of St. John of God, Prüfeninger Straße 86, D-93049 Regensburg, Germany

**Keywords:** Geriatrics, Outcomes research

## Abstract

Recently we introduced a modified Mini Nutritional Assessment (MNA) Short Form (MNA-SF) and Long Form (MNA-SF) with operationalization of the ‘mobility’ and ‘neuropsychological problems’ items of the MNA using scores on Barthel Index mobility item and Mini Mental State Examination and Geriatric Depression Scale scores. We have now evaluated the abilities of this modified MNA-SF and MNA-LF to predict mortality in comparison with the standard MNA-SF and MNA-LF and the Nutritional Risk Screening 2002 (NRS 2002) and the Malnutrition Universal Screening Tool (MUST). A prospective analysis was performed in 240 hospitalised geriatric patients aged ≥ 65 years. Malnutrition and/or malnutrition risk were assessed using the modified MNA-SF and MNA-LF, the standard MNA-SF and MNA-LF, and the NRS 2002 and MUST. The modified MNA-SF and MNA-LF and the standard MNA-SF and MNA-LF assessments (all *p* < 0.05), but not NRS 2002 or MUST (all *p* ≥ 0.05), predicted six-month and/or one-year mortality. Prediction of six-month and/or one-year mortality by the modified MNA-SF was comparable with predictions by the standard MNA-SF and MNA-LF (all *p* ≥ 0.05). The modified MNA-LF showed better prediction of six-month and one-year mortality than the standard MNA-SF and MNA-LF (all *p* < 0.05). The modified MNA-LF (all adjusted *p* < 0.05), but none of the other instruments (all adjusted *p* ≥ 0.05), predicted six-month and one-year mortality independently of age, sex, frailty, comorbidity and ADL disability burden. The modified MNA-SF and MNA-LF emerged as potentially valuable tools for predicting mortality in patients hospitalised on geriatric wards.

## Introduction

Malnutrition is an acute, subacute or chronic state of overnutrition or undernutrition which can include an inflammatory component and impacts body composition and function^1,2^. Malnutrition is often found in individuals with impairments to body functions such as dysphagia^3^, immobility^4^, depression^5^, limited perception of hunger and thirst, polypharmacy^6,7^, and acute and chronic diseases^7,8^. Patients on geriatric wards frequently show a high prevalence of malnutrition or are at high risk of malnutrition^7,9–11^. Malnourished patients have higher risks of sarcopenia^12,13^, frailty^14,15^, morbidity^8,16^ and mortality^8,16–20^. Previous analysis has shown that malnourished geriatric patients experience clinical endpoints, including mortality, more often or, strictly speaking, earlier than well-nourished people of the same age^8,16,17^. There are therefore advantages in detecting malnutrition and the risk of malnutrition early to drive timely dietary interventions in patients on geriatric wards.

Several different screening tools for malnutrition and/or the risk of malnutrition have been introduced. These include the Mini Nutritional Assessment (MNA) Short Form (MNA-SF)^21,22^ and Long Form (full MNA or MNA-LF)^23^, the Nutritional Risk Screening 2002 (NRS 2002)^24,25^, and the Malnutrition Universal Screening Tool (MUST)^26^, among others. Of these, the MNA^21–23^ and in particular the MNA-SF^21,22^ may be the instruments of choice in all geriatric healthcare settings, including patients on geriatric wards^27^. However, one drawback is that some items on the MNA-SF and MNA-LF, particularly ‘mobility’ and ‘neuropsychological problems’, depend on subjective interpretation by the examiner^28^. This suggests that from the clinical point of view a more standardized and objective implementation of these two MNA items would be advantageous^28^.

In Germany, patients hospitalised for acute care on geriatric wards are routinely evaluated in relation to 5 dimensions of a comprehensive geriatric assessment (CGA^29^) by the multidisciplinary geriatric team^30^. These five dimensions are cognition, emotion, mobility, activities of daily living (ADL), and social situation^29^. The Mini Mental State Examination (MMSE), Geriatric Depression Scale (GDS), the Timed Up and Go Test (TUG), Barthel Index, and a 5-item questionnaire addressing the patient’s social situation are frequently applied as assessment instruments^29^. As the prevalence or risk of malnutrition in patients hospitalised on geriatric wards is high, ever more geriatricians in Germany prefer to include evaluation of nutritional status in their assessments. With the aim of providing an objective and efficient approach to evaluating the nutritional status of older in-patients on geriatric wards, there may be value in using results from the mobility item of the Barthel Index and MMSE and GDS scores to operationalize the ‘mobility’ and ‘neuropsychological problems’ items in parallel with the other standard items of the MNA-SF and MNA-LF^28^.

We have recently introduced a modified MNA in which the ‘mobility’ and ‘neuropsychological problems’ items of the MNA are operationalized on the basis of results from the mobility item of the Barthel Index and MMSE and GDS scores^28^. We have reported evaluation of the completion rate, prevalence, and agreement with respect to categorisation of nutritional status determined using the modified MNA-SF and MNA-LF in comparison with the NRS 2002 instrument in geriatric inpatients in a cross-sectional analysis^28^. However, there are no data on the predictive ability of the modified MNA-SF and MNA-LF for adverse clinical outcomes such as mortality. Of note, the standard MNA-SF and MNA-LF have repeatedly been found to have predictive power for mortality^31–36^. The ability to predict mortality can therefore be regarded as a characteristic feature of the MNA-SF and MNA-LF. We therefore believe there is value in determining whether or not the modified MNA-SF and MNA-LF also have predictive power for mortality and whether or not the hypothesised predictive power for mortality of the modified MNA-SF and MNA-LF is comparable to that of the standard MNA-SF and MNA-LF.

In the study presented here, we now aimed to analyse the abilities of the modified MNA-SF and MNA-LF^28^ to predict six-month and one-year mortality in hospitalised geriatric patients. Moreover, we aimed to compare the predictive abilities of the modified MNA-SF and MNA-LF^28^ for mortality in comparison with those of the standard MNA-SF and MNA-LF^21–23^, NRS 2002^24,25^ and MUST^26^ in this group of patients.

## Materials and Methods

### Study design and population

The study was a prospective longitudinal analysis in patients hospitalised on the geriatric wards of the Department of Internal Medicine III (Medicine of Ageing), Malteser Waldkrankenhaus St. Marien, Erlangen, Germany. The study population consisted of 240 patients. Patients were enrolled in the study programme between October 2015 and March 2016. Inclusion criteria were age ≥ 65 years, willingness and provision of consent for all relevant medical data to be held after baseline examination, even should death occur, and provision of consent to contact relatives, legal guardians, general practitioners, and the local town authority for residents/inhabitants to obtain information on current place of residence and living status (alive/deceased). Patients were evaluated in relation to malnutrition and risk of malnutrition by a single trained investigator (LB) during hospital stays on geriatric wards (baseline examination). Comprehensive geriatric assessment was performed in all 240 study participants by the geriatric team (including physical therapists, psychologists, occupational therapists, speech therapists nurses, and others) by applying routine comprehensive geriatric assessment instruments (Mini Mental State Examination^37^, Geriatric Depression Scale^38^, Timed Up and Go Test^39^, Barthel Index^40^, social situation assessment using a 5-item questionnaire^41^, frailty phenotype^42^, Cumulative Illness Rating Scale for Geriatrics^43^, and others). Patients were followed up six months and one year after baseline examination. Patients, patients’ relatives or legal guardians, and general practitioners were contacted by telephone six months and one year after baseline examination to obtain pertinent follow-up data relating to medical endpoints such as death due to any cause (including exact date of death). The study was approved by the Ethics Committee of the Friedrich-Alexander University, Erlangen-Nuremberg, Germany and complied with currently applicable laws. Informed written consent by the participant or a legal guardian was obtained before inclusion of patients into the study.

### Malnutrition screening tools

#### Standard mini nutritional assessment short form (MNA-SF) and mini nutritional assessment long form (MNA-LF)

In the early 1990s Guigoz *et al*. developed and introduced the 18-item MNA (full MNA, or MNA-LF) as a malnutrition screening instrument^23^. In 1996, Rubenstein *et al*. developed and validated a short form (MNA-SF) including only six of the 18 items of the MNA-LF^21^. In 2009, Kaiser *et al*. showed that the ‘Body Mass Index (BMI)’ item of the MNA-SF and MNA-LF could be replaced by calf circumference when BMI could not be determined^22^. The six items of the standard MNA-SF were: ‘decline of food intake over the past three months’ (item A), ‘weight loss during the last three months’ (item B), ‘mobility’ (item C), ‘psychological stress or acute disease during the last three months’ (item D), ‘neuropsychological problems’ (item E), and ‘Body Mass Index’ (item F1), replacing BMI with ‘calf circumference (CC) in cm’ when BMI could not be determined (item F2)^21,22^. The items in the standard MNA-LF included the six items of the MNA-SF, along with twelve additional items: ‘independent living situation (not living in nursing home)’ (item G), ‘intake of more than 3 prescription drugs per day’ (item H), ‘pressure sores or skin ulcers’ (item I), ‘number of full daily meals’ (item J), ‘consumption markers for protein intake’ (item K), ‘consumption of two or more portions of fruit or vegetables per day’ (item L), ‘fluid intake’ (item M), ‘mode of feeding’ (item N), ‘self-assessment of nutritional status’ (item O), ‘self-assessment of health status in comparison with people of the same age’ (item P), ‘mid- arm circumference (MAC) in cm’ (item Q), and ‘calf circumference (CC) in cm’ (item R)^23^. Patients’ nutritional status in the standard MNA-SF and MNA-LF was classified as ‘normal nutritional status’, ‘at risk of malnutrition’, and ‘malnutrition’ on the basis of criteria described in detail elsewhere^21–23^.

#### Modified mini nutritional assessment short form (MNA-SF) and mini nutritional assessment long form (MNA-LF)

In the modified MNA-SF and MNA-LF, item C ‘mobility’ of the standard MNA-SF and MNA-LF was operationalized on the basis of the score on the mobility item of the Barthel Index. In brief, the categories “bed or chair bound”, “able to move in the ward” and “able to leave the ward” of item C, ‘mobility’, of the standard MNA-SF and MNA-LF were operationalized in the modified MNA-SF and MNA-LF using scores of 0, 5 to 10, and 15 points on the mobility item of the Barthel Index respectively^28^. Item E, ‘neuropsychological problems’, of the modified MNA-SF and MNA-LF was operationalized on the basis of Mini Mental State Examination (MMSE) and Geriatric Depression Scale (GDS) scores^28^. Thus, the categories “severe dementia or depression”, “mild dementia”, and “no psychological problems” of item E, ‘neuropsychological problems’, of the standard MNA-SF and MNA-LF were operationalized in the modified MNA-SF and MNA-LF based on an MMSE score of <20 or a GDS score of 10–15 points, an MMSE score of 20–26 points or a GDS score of 6–9 points, and an MMSE score of 27–30 points or a GDS score < 6 points respectively^28^. In addition, the modified MNA-SF and/or MNA-LF also included A, B, D, and F-R of the standard MNA-SF and/or MNA-LF, using the standard implementation and scoring of these items of the standard MNA-SF and MNA-LF^21–23,28^. Like the standard MNA-SF and MNA-LF, the modified MNA-SF and MNA-LF classified patients’ nutritional status into ‘normal nutritional status, ‘at risk of malnutrition’, and ‘malnutrition’^21–23,28^.

#### Nutritional risk screening 2002 (NRS 2002)

The NRS 2002 consists of a prescreen and a final screen. The prescreen includes four questions: I) ‘is BMI < 20.5 kg/m²?’, II) ‘has the patient lost weight within the last three months?’; III) ‘has the patient had a reduced dietary intake in the last week?’; and IV) ‘is the patient severely ill (i.e., in intensive therapy)?’, which have yes/no answers^24,25^. If the prescreen is positive, i.e., at least one of the four prescreening questions generates a ‘yes’ answer, a final and more comprehensive screening is performed and includes three dimensions: I) ‘nutritional, anthropometric and clinical status’; II) ‘disease severity; and III) ‘age of 70 years or older’^24,25^. Patients are identified as ‘no risk for malnutrition’ or ‘at risk of malnutrition’ on the NRS 2002, as described in detail elsewhere^24,25^.

#### Malnutrition universal screening tool (MUST)

The MUST addresses three items: ‘body mass index’, ‘undesired weight loss between the last three to six-months’, and ‘acute disease causing fasting for more than five days’^26,44^. Patients are identified as at ‘low risk’, ‘medium risk’, or ‘high risk of malnutrition’ on the MUST, as described in detail elsewhere^26,44^.

### Statistical analysis

Data were analysed in SPSS version 24 (IBM SPSS Statistics, Armonk, NY, USA). Results are presented in the text and/or tables as mean ± standard deviation and/or percentages. Groups of patients, i.e., patients who had died/survived to follow-up at six months and one year, were compared using the Mann-Whitney U-test or the chi-squared test as appropriate. The six-month and one-year mortality rates for the different categories defined by the malnutrition screening instruments were analysed by Kaplan Meier analysis. The log-rank test was used to differentiate the predictive power of the different categories defined by the malnutrition screening instruments in relation to six-month and one-year mortality. Cox proportional hazard models were run to analyse the hazard ratios for six-month and one-year mortality of each increment in category on the various malnutrition screening instruments. Hazard ratios (HRs) for six-month and one-year mortality of the various malnutrition screening instruments were adjusted for potential confounding factors (i.e., sex, age, frailty status, Cumulative Illness Rating Scale – Geriatrics (CIRS-G), and Barthel-Index score) and were considered both separately and together. Receivers operating characteristic (ROC) curves were used to estimate the area under the curves (AUC) for the various malnutrition screening tools, analysed as categorical variables, in relation to six-month and one-year mortality. The abilities of the various malnutrition screening tools to predict six-month and one-year mortality were compared by comparing the AUC of the malnutrition screening tools for six-month and one-year mortality using the Hanley and McNeil method^45^. Statistically significant differences were identified at *p* < 0.05.

## Results

### Clinical characteristics of the study cohort

The study cohort consisted of 240 patients hospitalised on geriatric wards (159 female and 81 male). The clinical characteristics of the overall study cohort and individuals stratified by death/survival at six months and one year are shown in Table 1. Follow-up data at six months were not available for one person. Of the remaining 239 for whom follow-up data were available at six month follow-up, 28 individuals (11.7%) had died. Among these 239 patients, 67.4% were female. Mean age was 82.5 ± 6.1 years, mean BMI was 24.8 ± 8.1 kg/m^2^, mean weight was 66.6 ± 22.3 kg, and mean height was 158.0 ± 30.7 cm. A total of 51.9% of these patients described weight loss of >4.5 kg in the last year, 76.2% had had falls, 36.0% had heart failure, 10.5% had myocardial infarcts, 21.8% had stroke, 17.6% had cancer, 34.3% had diabetes mellitus, 25.9% had pulmonary disease, 41.8% had kidney disease, 11.3% had constipation, 61.9% had urinary incontinence or bladder catheter, 16.3% had bowel incontinence, and 96.2% received more than 5 medications. Mean MMSE was 25.6 ± 3.9 points, GDS was 4.4 ± 2.7 points, Barthel Index was 68.8 ± 19.6 points, CIRS-G was 18.4 ± 5.4, 77.4% were frail on the Frailty Phenotype, and 65.3% had TUG > 19 sec or were unable to perform the TUG. Follow-up data at one year were not available for four persons, including the individual from whom follow-up data were not available at six months. Of the remaining 236 patients for whom follow-up data were available at one-year, 49 individuals (20.8%) had died. These 236 patients included 67.8% females, had an average age of 82.5 ± 6.1 years, a mean BMI of 24.9 ± 8.0 kg/m², a mean weight of 66.7 ± 22 kg, and a mean height of 158.0 ± 30.8 cm. A total of 51.3% of these 236 patients described weight loss >4.5 kg in the last year, 76.3% had had falls, 35.6% had heart failure, 10.2% had myocardial infarcts, 21.6% had stroke, 17.4% had cancer, 34.7% had diabetes mellitus, 25.8% had pulmonary disease, 41.9% had kidney disease, 11.4% had constipation, 61.4% had urinary incontinence or were catheterised, 15.3% had bowel incontinence, and 96.2% took more than five medications. Mean MMSE was 25.6 ± 4.0 points, GDS was 4.4 ± 2.7 points, Barthel Index was 67.2 ± 19.4 points, CIRS-G was 18.4 ± 5.4, 77.1% were frail on the Frailty Phenotype, and 64.8% of the patients had a TUG > 19 sec or were unable to perform the TUG. A total of 16.1% of these 236 patients were institutionalised.

**Table 1:** Clinical characteristics (baseline examination) of the study cohort and the two patient subgroups by using Mann- Whitney U-test or chi-squared test.

Clinical characteristics	All person (n = 240)	Persons who died during 6 month of follow-up (n = 28)	Persons who survived during 6 month of follow-up (n = 211)	P-value	Persons who died during 1 year of follow up (n = 49)	Persons who survived during 1 year of follow up (n = 187)	P-value
Age (years) mean (SD)	82.5 ± 6.1	83.4 ± 8.1	82.4 ± 5.8	0.136	82.9 ± 7.6	82.4 ± 5.7	0.329
Women, % (n)	67.5 (162)	50.0 (14)	69.7 (147)	0.037	55.1 (27)	71.1 (133)	0.033
Height (cm) mean (SD)	158.0 ± 31.0	153.0 ± 44.0	159.0 ± 29.0	0.772	159.0 ± 35.0	158.0 ± 30.0	0.274
Weight (kg) mean (SD)	66.5 ± 22.0	68.2 ± 20.0	66.4 ± 23.0	0.720	70.0 ± 23.0	65.9 ± 22.0	0.244
BMI (kg m^−2^), mean (SD)	24.8 ± 8.1	24.2 ± 8.4	24.9 ± 8.1	0.629	24.9 ± 8.3	24.9 ± 7.9	0.930
Weight loss > 4,5 kg in last year (%), (n)	51.7 (124)	64.3 (18)	50.2 (106)	0.006	61.2 (30)	48.7 (91)	0.035
Falls (%), (n)	75.8 (182)	71.4 (20)	76.8 (162)	0.533	69.4 (34)	78.1 (146)	0.203
Heart failure (%), (n)	36.3 (87)	57.1 (16)	33.2 (70)	0.013	51.0 (25)	31.6 (59)	0.011
Myocardial infarction (%), (n)	10.4 (25)	14.3 (4)	10.0 (21)	0.481	10.2 (5)	10.2 (19)	0.993
Stroke (%), (n)	21.7 (52)	17.9 (5)	22.3 (47)	0.594	18.4 (9)	22.5 (42)	0.536
Cancer (%), (n)	17.9 (43)	35.7 (10)	15.2 (32)	0.007	32.7 (16)	12.8 (24)	0.002
Diabetes mellitus (%), (n)	34.2 (82)	32.1 (9)	34.6 (73)	0.797	38.8 (19)	33.7 (63)	0.506
Pulmonary disease (%), (n)	26.3 (63)	35.7 (10)	24.6 (52)	0.209	26.5 (13)	25.7 (48)	0.902
Kidney disease (%), (n)	41.7 (100)	64.3 (18)	38.9 (82)	0.010	57.1 (28)	38.0 (71)	0.015
Constipation (%), (n)	11.7 (28)	7.1 (2)	11.8 (25)	0.460	6.1 (3)	12.8 (24)	0.189
Urinary incontinence or bladder catheter (%), (n)	62.1 (149)	75.0 (21)	60.2 (127)	0.129	73.5 (36)	58.3 (109)	0.052
Bowel incontinence (%), (n)	16.7 (40)	32.1 (9)	14.2 (30)	0.016	28.6 (14)	11.8 (22)	0.004
More than 5 medications (%), (n)	96.3 (231)	96.4 (27)	96.2 (203)	0.954	98.0 (48)	95.7 (179)	0.467
Mini- Mental State Examination, mean (SD)	25.6 ± 4.0	26.0 ± 3.2	25.5 ± 4.0	0.778	26.0 ± 3.15	25.5 ± 4.1	0.660
Geriatric Depression Scale, mean (SD)	4.4 ± 2.7	5.7 ± 3.3	4.2 ± 2.6	0.028	5.1 ± 2.9	4.2 ± 2.6	0.049
Barthel Index Score, mean (SD)	66.8 ± 20.0	48.9 ± 20.0	69.2 ± 18.2	<0.001	54.6 ± 19.2	70.5 ± 18.0	<0.001
Timed Up and Go Test > 19 seconds/ unable (%), (n)	65.4 (157)	92.9 (26)	61.6 (130)	0.001	91.8 (45)	57.8 (108)	<0.001
Institutionalized (%), (n) Frail by Frailty Phenotype (%), (n) Cumulative Illness Rating Scale, mean (SD)	16.7 (40) 77.5 (186) 18.4 ± 5.4	28.6 (8) 92.9 (26) 22.4 ± 4.6	14.7 (31) 75.4 (159) 17.9 ± 5.3	0.142 0.114 0.519	24.5 (12) 91.8 (45) 21.3 ± 5.8	13.9 (26) 73.3 (137) 17.6 ± 5.1	0.199 0.022 0.340

Assessment on both the modified MNA-SF and standard MNA-SF categorised 98.8% of the 240 study participants as “malnourished or at risk of malnutrition”. The proportions classified as malnourished and at risk for malnutrition differed: the modified MNA-SF identified 51.7% of individuals as “malnourished” and 47.1% as “at risk of malnutrition,” whereas the standard MNA-SF identified 56.7% of study participants as “malnourished” and 42.0% as “at risk of malnutrition.” The modified MNA-LF and standard MNA-LF categorised 97.5% and 97.9% patients as “malnourished or at risk of malnutrition”. The modified MNA-LF identified 37.5% as “malnourished” and 60.0% as “at risk of malnutrition”, while the standard MNA-LF classified 37.1% as “malnourished” and 60.8% as “at risk of malnutrition”.

### Comparison of mortality rates at six-month and one-year follow-up stratified by nutritional status as determined with the various malnutrition screening instruments

**Figure 1:**
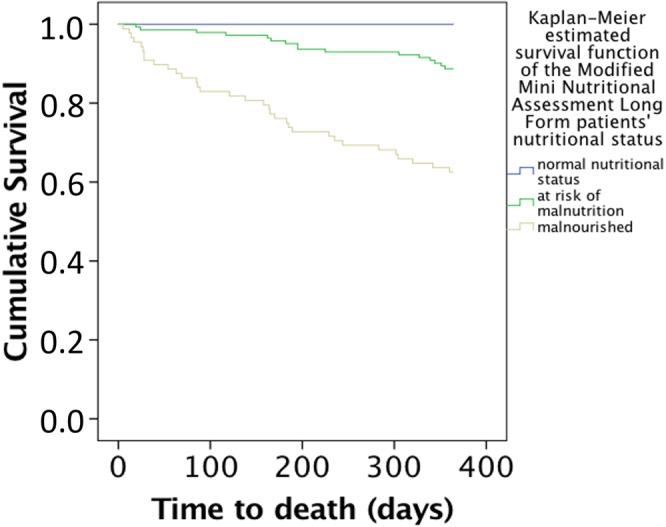
Kaplan-Meier estimated survival function of patients stratified by nutritional status as evaluated using the Modified Mini Nutritional Assessment Short Form, Log- Rank test: χ^2^ = 7.1, p-value 0.029.

**Figure 2:**
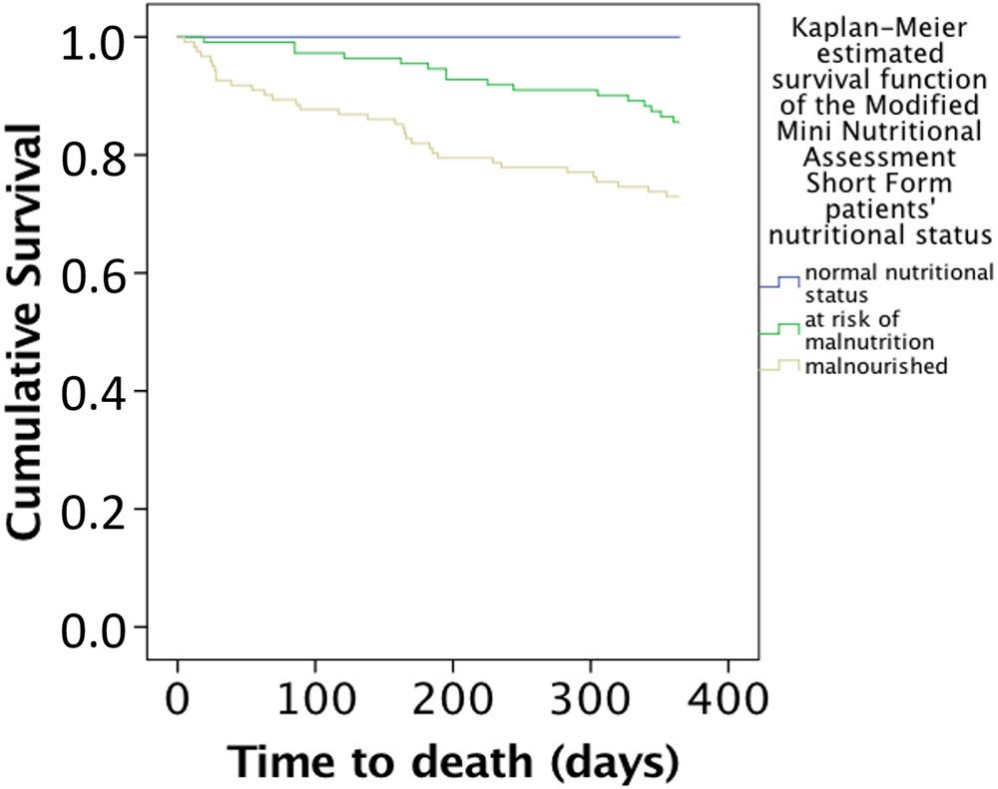
Kaplan-Meier estimated survival function of patients stratified by nutritional status as evaluated using the Modified Mini Nutritional Assessment Long Form, Log- Rank test: χ^2^ = 26.9, p-value < 0.001.

Mortality rates at six-month and one-year follow-up stratified by nutritional status on the various malnutrition screening instruments are given in Table 2 and Figs 1 and 2. Patients with adverse malnutrition status on the modified MNA-SF and MNA-LF and the standard MNA-SF and MNA-LF had higher mortality rates than individuals with better nutritional status (all *p* < 0.05) (see Table 2). Mortality rates for six-month mortality showed no statistically significant differences between patients with different malnutrition status on the NRS 2002 (*p* ≥ 0.05), though one-year mortality was higher in patients with adverse malnutrition status than in individuals with better nutritional status (all *p* < 0.05). Mortality rates showed no statistically significant differences between patients with different malnutrition status on the MUST for either six-month and/or one-year mortality (all *p* ≥ 0.05) (see Table 2)

**Table 2:** Mortality rates at six-month and one-year of follow- up according to the nutritional status of the different malnutrition screening instruments by using Kaplan Meier analyses.

	Persons with “not at risk of malnutrition and not malnourished” or “low risk” using MUST %,	Persons with “at risk of malnutrition” or “medium risk” using MUST %	Persons with “being malnourished” or “high risk” using MUST %	p- value
**Six-month mortality**				
modified MNA- SF	0.0% (0)	5.4% (6)	17.7% (22)	0.010
modified MNA- LF	0.0% (0)	4.9% (7)	23.3% (21)	<0.001
MNA- SF	0.0% (0)	6.0% (6)	16.2% (22)	0.046
MNA- LF	0.0% (0)	6.9% (10)	20.2% (18)	0.006
NRS 2002	5.6% (2)	12.8% (26)	—	0.212
MUST	8.5% (10)	9.1% (5)	19.4% (13)	0.070
**One-year mortality**				
modified MNA- SF	0.0% (0)	14.4% (16)	27.0% (33)	0.040
modified MNA- LF	0.0% (0)	11.3% (16)	20.8% (33)	<0.001
MNA- SF	0.0% (0)	14.0% (14)	26.3% (35)	0.048
MNA- LF	20.0% (1)	13.9% (20)	32.2% (28)	0.004
NRS 2002	8.3% (3)	23.0% (46)	—	0.046
MUST	16.2% (19)	20.4% (11)	29.2% (19)	0.117

### Prediction of six-month and one-year mortality

The abilities of the various malnutrition screening instruments to predict six-month and one-year mortality are shown and compared in Table 3. The modified MNA-SF and MNA-LF and the standard MNA-SF and MNA-LF, but not the NRS 2002 or the MUST, were able to predict six-month and one-year mortality (see Table 3). The ability of the modified MNA-SF to predict six-month mortality was better than that of the NRS 2002 but showed no differences compared with the modified MNA-LF, standard MNA-SF, MNA-LF and MUST. The ability of the modified MNA-SF to predict one-year mortality showed no difference compared with the standard MNA-SF, MNA-LF, NRS 2002, and MUST and was worse than that of the modified MNA-LF. With the exception of the modified MNA-SF, the modified MNA-LF yielded better predictions of six-month mortality than all other malnutrition screening instruments evaluated in this study. The modified MNA-LF gave better prediction of one-year mortality than all other malnutrition screening tools evaluated here.

**Table 3:** Ability and comparison of the ability of the different malnutrition screening tools to predict six-month and one-year mortality by analysing Receivers operating characteristic (ROC).

	AUC (95% CI)	P- Value	P- Value, AUC 1 vs AUCs 2,3,4,5 and 6	P- Value, AUC 2 vs AUCs 1, 3, 4, 5 and 6	P- Value, AUC 3 vs AUCs 1, 2, 4, 5 and 6	P- Value, AUC 4 vs AUCs 1, 2, 3, 5 and 6	P- Value, AUC 5 vs AUCs 1, 2, 3, 4 and 6	P- Value, AUC 6 vs AUCs 1, 2, 3, 4 and 5
**Six-month mortality**								
1. Modified MNA-SF	0.653 (0.553–0.753)	0.009	—	0.115	0.249	0.472	0.038	0.184
2. Modified MNA-LF	0.715 (0.617–0.813)	< 0.001	0.115	—	0.022	<0.001	0.006	0.033
3. MNA-SF	0.624 (0.522–0.726)	0.033	0.252	0.023	—	0.253	0.125	0.383
4. MNA-LF	0.657 (0.550–0.764)	0.007	0.472	<0.001	0.253	—	0.058	0.211
5. NRS 2002	0.545 (0.437–0.652)	0.441	0.038	0.006	0.085	0.058	—	0.164
6. MUST	0.607 (0.491–0.723)	0.066	0.184	0.033	0.383	0.211	0.164	—
**One- year mortality**								
1. Modified MNA-SF	0.601 (0.515–0.688)	0.029	—	0.016	0.457	0.300	0.208	0.352
2. Modified MNA-LF	0.695 (0.612–0.778)	<0.001	0.016	—	0.005	<0.001	0.006	0.010
3. MNA-SF	0.597 (0.511–0.684)	0.036	0.457	0.005	—	0.243	0.208	0.400
4. MNA-LF	0.626 (0.535–0.716)	0.007	0.300	<0.001	0.243	—	0.121	0.210
5. NRS 2002	0.558 (0.472–0.643)	0.215	0.197	0.008	0.204	0.121	—	0.296
6. MUST	0.585 (0.494–0.676)	0.067	0.352	0.012	0.366	0.210	0.300	—

### Hazard Ratios and adjusted Hazard ratios for each increment in category on the various malnutrition screening tools in relation to six-month and one-year mortality

Unadjusted and adjusted HR for each increment in category on the different malnutrition screening instruments are given in Table 4. Each increment in category on the modified MNA-SF and MNA-LF and the standard MNA-SF and MNA-LF and the MUST, but not the NRS 2002, increased the risk for six-month and one-year mortality (see Table 4). In an adjusted model taking age and sex taking into account, each increment in category of the modified MNA-SF, the modified MNA-LF, the standard MNA-SF, the standard MNA-LF, and the MUST, but not NRS 2002, was associated with an increased risk for six-month and one-year mortality (see Table 4). In a further adjusted model taking several potential confounding factors into account (age, sex, frailty status according to the frailty phenotype, Cumulative Illness Rating Scale for Geriatrics (CIRS-G), and Barthel Index score), each increment in category on the modified MNA-LF, but not on the modified MNA-SF, standard MNA-SF, standard MNA-LF, NRS 2002, or MUST, was associated with an increased risk for six-month and one-year mortality (see Table 4).

**Table 4:** Hazard Ratios and adjusted Hazard ratios for each increment in category of the different malnutrition screening tools in relation to six-month and one-year mortality.

Malnutrition screening tool	HR (95% CI)	P- Value	Adjusted HR* (95% CI)	P- Value	Adjusted HR** (95% CI)	P- Value
**Six-month mortality**						
Modified MNA- SF	3.642 (1.489–8.908)	0.005	3.382 (1.380–8.292)	0.008	1.803 (0.676–4.812)	0.239
Modified MNA- LF	5.441 (2.332–12.692)	<0.001	5.451 (2.335–12.725)	<0.001	2.928 (1.1149–7.643)	0.024
MNA- SF	2.940 (1.206–7.168)	0.018	2.631 (1.076–6.434)	0.034	1.206 (0.437–3.328)	0.718
MNA- LF	3.318 (1.550–7.101)	0.002	3.146 (1.469–6.739)	0.003	1.508 (0.635–3.581)	0.352
NRS 2002	2.412 (0.573–10.163)	0.230	2.391 (0.567–10.077)	0.235	1.787 (0.415–7.691)	0.436
MUST	1.582 (1.030–2.429)	0.036	1.605 (1.041–2.475)	0.032	1.434 (0.925–2.221)	0.107
**One-year mortality**						
Modified MNA- SF	2.179 (1.212–3.916)	0.009	2.047 (1.137–3.685)	0.017	1.198 (0.629–2.282)	0.582
Modified MNA- LF	4.161 (2.309–7.497)	<0.001	4.179 (2.318–7.534)	<0.001	2.644 (1.388–5.035)	0.003
MNA- SF	2.159 (1.176–3.965)	0.013	2.011 (1.095–3.693)	0.024	1.111 (0.564–2.187)	0.760
MNA- LF	2.446 (1.412–4.239)	0.001	2.365 (1.365–4.097)	0.002	1.349 (0.731–2.490)	0.338
NRS 2002	3.015 (0.938–9.696)	0.064	3.017 (0.938–9.702)	0.064	2.565 (0.790–8.328)	0.117
MUST	1.418 (1.028–1.957)	0.033	1.427 (1.032–1.974)	0.032	1.353 (0.974–1.879)	0.072

*Cox proportional hazard model considering age and sex.

**Cox proportional hazard model considering age, sex, Frailty Phenotype, CIRS-G and Barthel Index Score.

## Discussion

The major finding in the present study was that the modified MNA-SF and the standard MNA-SF did not differ in the ability to predict six-month and one-year mortality in our cohort of hospitalised geriatric patients. This indicates that the power of the modified MNA-SF to predict six-month and one-year mortality may be comparable with that of the standard MNA-SF in this group of patients. Thus, the modified MNA-SF emerges as a potentially valuable tool for predicting mortality in those hospitalised geriatric patients in whom the mobility item of the Barthel Index, MMSE and GDS is routinely applied during standard comprehensive geriatric assessments.

A further major finding of this study is that the modified MNA-LF displayed better prediction of six-month and one-year mortality than the standard MNA-LF. This indicates that the modification of the two items - ‘mobility’ and ‘neuropsychological problems’ - in the modified MNA-LF may improve the ability of this malnutrition screening instrument to predict mortality in hospitalised geriatric patients. Moreover, with the exception of the modified MNA-SF (the modified MNA-LF and modified MNA-SF did not differ in the ability to predict six-month mortality), the modified MNA-LF yielded better predictions of six-month and one-year mortality than all other malnutrition screening instruments evaluated here. Similarly, Kiesswetter *et al*. reported that the MNA-LF gave better prediction of one-year mortality than the MNA-SF in a cohort of 309 older adults aged ≥ 65 years receiving home care^31^. In a study of 246 institutionalised individuals aged 76.5 ± 11 years, the MNA-LF had higher predictive value for survival of well-nourished participants then the other malnutrition screening instruments (MNA-SF, NRS 2002 and MUST)^32^. In contrast, a Taiwanese prospective cohort study of 2872 participants aged ≥ 65 years demonstrated that the MNA-SF was comparable or even marginally superior than the MNA-LF in predicting four-year mortality^33^. However, and of relevance in the busy clinical setting at geriatric wards, it is of note that administration of the modified MNA-LF is more time-consuming than the modified MNA-SF.

In the present study, all the different MNA versions, i.e., the modified MNA-SF, modified MNA-LF, standard MNA-SF, and standard MNA-LF, were able to predict six-month and one-year mortality. In contrast, in this study the NRS 2002 and the MUST, did not reveal such an ability to predict six-month and or one-year mortality. Diekmann *et al*. previously reported that the MNA had greater predictive power than the NRS 2002 and MUST for survival in a prospective longitudinal analysis with a follow-up period of up to one year in 200 nursing-home residents^34^. Koren-Hakim *et al*. reported that the MNA-SF, but not the NRS 2002 or MUST, was able to predict mortality during a 36-month follow-up period with measurement of nutritional status in 215 older people undergoing hip fracture surgery, with a mean age of 83.9 ± 6.09 years^35^. Donini *et al*. reported that the MNA-LF and MNA-SF had higher predictive value for mortality than the NRS 2002 and MUST in a study cohort of 246 institutionalised participants aged 76.5 ± 11 years^32^. In contrast, a Brazilian study of a cohort of 705 participants, which included 169 geriatric patients aged ≥ 65 years, found that the abilities of the MNA-SF and NRS 2002 to predict complications, prolonged hospital stay, and mortality in older patients were comparable^36^. Holst *et al*. studied a cohort of 233 hospitalised geriatric patients and found that neither the MNA-LF, nor the NRS 2002, nor the MUST was able to predict one-year mortality^46^.

Among the different malnutrition screening instruments evaluated here, only the modified MNA-LF displayed independent predictive value for six-month and one-year mortality after adjustment of the analysis for potential confounding factors, including age, sex, frailty status, comorbidity, and ADL disability burden. There might be an interaction between age, sex, frailty status, comorbidity and ADL disability on the one hand and malnutrition and the risk of malnutrition on the other in hospitalised geriatric patients with, along with each of these conditions, a potential impact on the mortality risk. Among the malnutrition screening instruments evaluated here, the modified MNA-LF may be the most objective and complex, and probably allows the predictive value of malnutrition and risk for malnutrition to be discriminated from the contributions of age, sex, frailty status, comorbidity and ADL disability to prediction of mortality. Nevertheless, with the exception of the NRS 2002, all the malnutrition screening instruments evaluated here had predictive power for six-month and one-year mortality after adjustment of the analysis for age and sex only. In line with the findings reported here, several other authors have reported that malnutrition has independent predictive value for mortality in different settings. Jiang *et al*. reported studies of a cohort of 437 patients with a median age of 81.0 (74.5–84.0) years in an acute geriatric ward showing that malnutrition as defined by the European Society for Clinical Nutrition and Metabolism (ESPEN) criteria, but not by the MNA, was an independent predictor of three-year mortality^47^. In a study of 131 patients aged ≥ 60 years, the Geriatric Nutritional Risk Index (GNRI) predicted three- and six-month mortality independent of potential confounding factors, including age, sex, and cancer^48^. In a retrospective study of 1170 participants aged ≥ 60 years living in a community dwelling setting, malnutrition was found to be an independent risk factor for seven-year mortality^49^. A prospective cohort study of 164 emergency department patients aged ≥ 75 years showed that malnutrition was a strong independent risk factor for short-term mortality in geriatric patients^50^. Liu *et al*. reported a prospective observational study including diabetes patients aged ≥ 65 years with a 2.8-year follow-up which showed that malnutrition was an independent predictor of mortality^51^. Another study of a cohort of 1306 participants aged ≥ 75 years showed that malnutrition as assessed on the MNA-SF was independently related to six-month mortality^52^. Correia *et al*. also noted that malnutrition was an independent risk factor of mortality^16^. Lilamand *et al*. reported that the MNA-SF predicted one-year mortality independently of potential confounding factors in a nursing home setting in a study of 773 older people with a mean age of 86.2 ± 7.5 years^53^. It should be noted that the setting on hospital geriatric wards may not be comparable to the setting in the community/home or nursing homes.

The six-month and one-year mortality rates in the present study in hospitalised geriatric patients-11.7% and 20.8% - are in line with mortality rates reported from other studies by ourselves and other authors in hospitalised geriatric patients. We have previously reported that 15.4% of 306 study participants died during the first six-month post-baseline examination in a cohort of hospitalised geriatric patients^54,55^. Dent *et al*. found a six-month mortality rate of 16% in 172 hospitalised geriatric patients^56^. A study by Drame *et al*. of 1306 acute hospitalised study participants aged ≥ 75 years found that a six-month mortality rate of 24.4%^52^. One of our own earlier studies yielded a one-year mortality of 20.3% in 304 hospitalised geriatric patients^57,58^. A study of 2033 geriatric in-patients recorded a one-year mortality of 24.9%^59^. Holst *et al*. reported a one-year mortality rate of 27% in 233 hospitalised geriatric patients^46^.

The mortality rates of individuals with malnutrition or at risk for malnutrition as evaluated by the modified MNA-SF and modified MNA-LF in the present study are similar to those in our previous studies using the malnutrition screening instruments identified above in hospitalised geriatric patients^28^.

This study has a number of strengths. It is the first prospective longitudinal analysis dissecting the ability of the modified MNA (modified MNA-SF and modified MNA-LF) as compared with the standard MNA-SF, MNA-LF, NRS 2002 and MUST to predict six-month and one-year mortality. Furthermore, we adjusted the analysis for potential confounding factors to analyse whether malnutrition as classified by the different screening instruments has independent predictive value for six-month and/or one-year mortality. Follow-up data were obtained at two time points (six months and one year post-baseline). The rate of loss to follow-up was very low. Only 0.4% of the 240 study participants could not be reached at the six-months and 1.7% at one year. Previous studies showed comparable percentages of loss to follow up at six months or one year^54,57,58,60^.

On average it takes 5–10 minutes to perform the MNA-SF and 10–20 minutes to perform the MNA-LF depending on individual patient characteristics. In the case the MMSE, GDS and Barthel Index are routinely applied in terms of the comprehensive geriatric assessment at the geriatric ward the assessor can save approximately up to five minutes by using the score of the MMSE, GDS and mobility item of the Barthel Index to operationalize the modified MNA-SF and modified MNA-LF. Clearly, in the case the MMSE, GDS and Barthel Index are not routinely applied in terms of the comprehensive geriatric assessment at the geriatric ward it would take longer to operationalize the modified MNA-SF and modified MNA-LF in comparison to the standard MNA-SF and standard MNA-LF as the MMSE, GDS and mobility item of the Barthel Index have to be performed in addition.

The study also has some limitations. The caring geriatric teams were not blinded to the results of all these scores of the comprehensive geriatric assessment tools at the time of admission and may have altered their care plans on the basis of these data. This may have altered mortality risk potentially confounding the observed results. It was a single-centre study of hospitalised patients on geriatric wards. It may therefore be misleading to extrapolate the findings to the geriatric wards of any other hospital or other clinical setting. All study participants were Caucasian, so the study results may not be transferable to other ethnic groups.

In conclusion, the modified MNA-SF and standard MNA-SF showed comparable abilities to predict six-month and one-year mortality in hospitalised geriatric patients. With the exception of the modified MNA-SF (there were no differences in the abilities of the modified MNA-LF and modified MNA-SF to predict six-month mortality), the modified MNA-LF gave better predictions of six-month and one-year mortality than all the other malnutrition screening instruments evaluated here. The various MNA versions evaluated in this study, i.e., the modified MNA-SF and MNA-LF and the standard MNA-SF and MNA-LF, and the MUST, but not the NRS 2002, were able to predict six-month and one-year mortality after adjustment of the analysis for age and sex. The modified MNA-LF, but none of the other malnutrition screening instruments evaluated here, had independent predictive value for six-month and one-year mortality after adjustment of the analysis for age, sex, frailty status, comorbidity and ADL disability burden. The modified MNA-SF and modified MNA-LF therefore emerge as potentially valuable tools for predicting mortality in hospitalised geriatric patients in whom the Barthel Index, MMSE and GDS are routinely applied in the course of standardised comprehensive geriatric assessment.

## Data Availability

The datasets generated and/or analysed during the current study are available from the corresponding author on reasonable request.

## References

[1] American Society for Parenteral and Enteral Nutrition (A.S.P.E.N.) Board of Clinical Practice Committee. Definition of terms, style, and conventions used in A.S.P.E.N. (2010).

[2] Mueller C, Compher C, Ellen DMASPEN (2011). clinical guidelines: Nutrition screening. assessment, and intervention in adults. JPEN.

[3] Eglseer D, Halfens RJG, Schols J, Lohrmann C (2018). Dysphagia in Hospitalized Older Patients: Associated Factors and Nutritional Interventions. J Nutr Health Aging.

[4] Konturek PC, Herrmann HJ, Schink K, Neurath MF, Zopf Y (2015). Malnutrition in Hospitals: It Was, Is Now, and Must Not Remain a Problem!. Med Sci Monit.

[5] Morley JE, Kraenzle D (1994). Causes of weight loss in a community nursing home. J Am Geriatr Soc.

[6] Little MO (2018). Updates in nutrition and polypharmacy. Curr Opin Clin Nutr Metab Care.

[7] Pirlich M (2006). The German hospital malnutrition study. Clin Nutr.

[8] Norman K, Pichard C, Lochs H, Pirlich M (2008). Prognostic impact of disease-related malnutrition. Clin Nutr.

[9] Kaiser MJ (2010). Frequency of malnutrition in older adults: a multinational perspective using the mini nutritional assessment. J Am Geriatr Soc.

[10] Schrader E, Grosch E, Bertsch T, Sieber CC, Volkert D (2016). Nutritional and Functional Status in Geriatric Day Hospital Patients - MNA Short Form Versus Full MNA. J Nutr Health Aging.

[11] Volkert D, Saeglitz C, Gueldenzoph H, Sieber CC, Stehle P (2010). Undiagnosed malnutrition and nutrition-related problems in geriatric patients. J Nutr Health Aging.

[12] Rolland Y (2008). Sarcopenia: its assessment, etiology, pathogenesis, consequences and future perspectives. J Nutr Health Aging.

[13] Volkert D (2011). The role of nutrition in the prevention of sarcopenia. Wien Med Wochenschr.

[14] Bonnefoy M (2015). Frailty and nutrition: searching for evidence. J Nutr Health Aging.

[15] Dorner TE (2014). Association between nutritional status (MNA(R)-SF) and frailty (SHARE-FI) in acute hospitalised elderly patients. J Nutr Health Aging.

[16] Correia MI, Waitzberg DL (2003). The impact of malnutrition on morbidity, mortality, length of hospital stay and costs evaluated through a multivariate model analysis. Clin Nutr.

[17] Caccialanza R, Cereda E, Klersy C (2011). Malnutrition, age and inhospital mortality. Cmaj.

[18] Asiimwe SB, Muzoora C, Wilson LA, Moore CC (2015). Bedside measures of malnutrition and association with mortality in hospitalized adults. Clin Nutr.

[19] Ulger Z (2013). Malnutrition in Turkish nursing homes: a correlate of short term mortality. J Nutr Health Aging.

[20] Kang MC (2018). Prevalence of Malnutrition in Hospitalized Patients: a Multicenter Cross-sectional Study. J Korean Med Sci.

[21] Rubenstein LZ, Harker JO, Salva A, Guigoz Y, Vellas B (2001). Screening for undernutrition in geriatric practice: developing the short-form mini-nutritional assessment (MNA-SF). J Gerontol A Biol Sci Med Sci.

[22] Kaiser MJ (2009). Validation of the Mini Nutritional Assessment short-form (MNA-SF): a practical tool for identification of nutritional status. J Nutr Health Aging.

[23] Guigoz Y, Vellas B, Garry PJ (1996). Assessing the nutritional status of the elderly: The Mini Nutritional Assessment as part of the geriatric evaluation. Nutr Rev.

[24] Kondrup J, Rasmussen HH, Hamberg O, Stanga Z (2003). Nutritional risk screening (NRS 2002): a new method based on an analysis of controlled clinical trials. Clin Nutr.

[25] Kondrup J, Allison SP, Elia M, Vellas B, Plauth M (2003). ESPEN guidelines for nutrition screening 2002. Clin Nutr.

[26] Orlandoni P (2017). Malnutrition upon Hospital Admission in Geriatric Patients: Why Assess It?. Front Nutr.

[27] Cederholm T (2017). ESPEN guidelines on definitions and terminology of clinical nutrition. Clin Nutr.

[28] Christner S (2016). Evaluation of the nutritional status of older hospitalised geriatric patients: a comparative analysis of a Mini Nutritional Assessment (MNA) version and the Nutritional Risk Screening (NRS 2002). J Hum Nutr Diet.

[29] Rockwood Kenneth, Silvius James L., Fox Roy A. (1998). Comprehensive geriatric assessment. Postgraduate Medicine.

[30] Applegate W, Deyo R, Kramer A, Meehan S (1991). Geriatric evaluation and management: current status and future research directions. J Am Geriatr Soc.

[31] Kiesswetter E (2014). Prognostic differences of the Mini Nutritional Assessment short form and long form in relation to 1-year functional decline and mortality in community-dwelling older adults receiving home care. J Am Geriatr Soc.

[32] Donini LM (2016). Mini-Nutritional Assessment, Malnutrition Universal Screening Tool, and Nutrition Risk Screening Tool for the Nutritional Evaluation of Older Nursing Home Residents. J Am Med Dir Assoc.

[33] Wang JY, Tsai AC (2013). The short-form mini-nutritional assessment is as effective as the full-mini nutritional assessment in predicting follow-up 4-year mortality in elderly Taiwanese. J Nutr Health Aging.

[34] Diekmann R (2013). Screening for malnutrition among nursing home residents - a comparative analysis of the mini nutritional assessment, the nutritional risk screening, and the malnutrition universal screening tool. J Nutr Health Aging.

[35] Koren-Hakim T (2016). Comparing the adequacy of the MNA-SF, NRS-2002 and MUST nutritional tools in assessing malnutrition in hip fracture operated elderly patients. Clin Nutr.

[36] Raslan M (2010). Comparison of nutritional risk screening tools for predicting clinical outcomes in hospitalized patients. Nutrition.

[37] Folstein MF, Folstein SE, McHugh PR (1975). Mini-mental state. A practical method for grading the cognitive state of patients for the clinician. J Psychiatr Res.

[38] Yesavage JA (1982). Development and validation of a geriatric depression screening scale: a preliminary report. J Psychiatr Res.

[39] Podsiadlo D, Richardson S (1991). The timed “Up & Go”: a test of basic functional mobility for frail elderly persons. J Am Geriatr Soc.

[40] Mahoney FI, Barthel DW (1965). Functional Evaluation: The Barthel Index. Md State Med J.

[41] Nikolaus T, Specht-Leible N, Bach M, Oster P, Schlierf G (1994). Social aspects in diagnosis and therapy of very elderly patients. Initial experiences with a newly developed questionnaire within the scope of geriatric assessment. Z Gerontol.

[42] Rockwood K, Andrew M, Mitnitski A (2007). A comparison of two approaches to measuring frailty in elderly people. J Gerontol A Biol Sci Med Sci.

[43] Linn BS, Linn MW, Gurel L (1968). Cumulative illness rating scale. J Am Geriatr Soc.

[44] Stratton RJ (2004). Malnutrition in hospital outpatients and inpatients: prevalence, concurrent validity and ease of use of the ‘malnutrition universal screening tool’ (‘MUST’) for adults. Br J Nutr.

[45] Hanley JA, McNeil BJ (1983). A method of comparing the areas under receiver operating characteristic curves derived from the same cases. Radiology.

[46] Holst M (2013). Nutritional screening and risk factors in elderly hospitalized patients: association to clinical outcome?. Scand J Caring Sci.

[47] Jiang J (2017). Predicting long-term mortality in hospitalized elderly patients using the new ESPEN definition. Sci Rep.

[48] Abd-El-Gawad WM, Abou-Hashem RM, El Maraghy MO, Amin GE (2014). The validity of Geriatric Nutrition Risk Index: simple tool for prediction of nutritional-related complication of hospitalized elderly patients. Comparison with Mini Nutritional Assessment. Clin Nutr.

[49] Ferreira LS (2011). Undernutrition as a major risk factor for death among older Brazilian adults in the community-dwelling setting: SABE survey. Nutrition.

[50] Gentile S (2013). Malnutrition: a highly predictive risk factor of short-term mortality in elderly presenting to the emergency department. J Nutr Health Aging.

[51] Liu GX (2017). Pilot study of the Mini Nutritional Assessment on predicting outcomes in older adults with type 2 diabetes. Geriatr Gerontol Int.

[52] Drame M (2012). Six-month outcome of elderly people hospitalized via the emergency department: the SAFES cohort. Rev Epidemiol Sante Publique.

[53] Lilamand M (2015). The Mini Nutritional Assessment-Short Form and mortality in nursing home residents–results from the INCUR study. J Nutr Health Aging.

[54] Ritt M, Jager J, Ritt JI, Sieber CC, Gassmann KG (2017). Operationalizing a frailty index using routine blood and urine tests. Clin Interv Aging.

[55] Ritt M (2016). A comparison of Frailty Indexes Based on a Comprehensive Geriatric Assessment for the Prediction of Adverse Outcomes. J Nutr Health Aging.

[56] Dent E, Chapman IM, Piantadosi C, Visvanathan R (2014). Performance of nutritional screening tools in predicting poor six-month outcome in hospitalised older patients. Asia Pac J Clin Nutr.

[57] Ritt M, Ritt JI, Sieber CC, Gassmann KG (2017). Comparing the predictive accuracy of frailty, comorbidity, and disability for mortality: a 1-year follow-up in patients hospitalized in geriatric wards. Clin Interv Aging.

[58] Ritt M, Bollheimer LC, Sieber CC, Gassmann KG (2016). Prediction of one-year mortality by five different frailty instruments: A comparative study in hospitalized geriatric patients. Arch Gerontol Geriatr.

[59] Pilotto A (2012). Comparing the prognostic accuracy for all-cause mortality of frailty instruments: a multicentre 1-year follow-up in hospitalized older patients. PLoS One.

[60] Ritt M (2015). Analysis of Rockwood et Al’s Clinical Frailty Scale and Fried et Al’s Frailty Phenotype as Predictors of Mortality and Other Clinical Outcomes in Older Patients Who Were Admitted to a Geriatric Ward. J Nutr Health Aging.

